# Insights into dynamic sliding contacts from conductive atomic force microscopy†

**DOI:** 10.1039/d0na00414f

**Published:** 2020-07-24

**Authors:** Nicholas Chan, Mohammad R. Vazirisereshk, Ashlie Martini, Philip Egberts

**Affiliations:** Department of Mechanical and Manufacturing Engineering, University of Calgary 2500 Drive NW Calgary Alberta T2N 1N4 Canada philip.egberts@ucalgary.ca; School of Engineering, University of California Merced 5200N Lake Road Merced California 95343 USA amartini@ucmerced.edu

## Abstract

Friction in nanoscale contacts is determined by the size and structure of the interface that is hidden between the contacting bodies. One approach to investigating the origins of friction is to measure electrical conductivity as a proxy for contact size and structure. However, the relationships between contact, friction and conductivity are not fully understood, limiting the usefulness of such measurements for interpreting dynamic sliding properties. Here, atomic force microscopy (AFM) was used to simultaneously acquire lattice resolution images of the lateral force and current flow through the tip–sample contact formed between a highly oriented pyrolytic graphite (HOPG) sample and a conductive diamond AFM probe to explore the underlying mechanisms and correlations between friction and conductivity. Both current and lateral force exhibited fluctuations corresponding to the periodicity of the HOPG lattice. Unexpectedly, while lateral force increased during stick events of atomic stick-slip, the current decreased exponentially. Molecular dynamics (MD) simulations of a simple model system reproduced these trends and showed that the origin of the inverse correlation between current and lateral force during atomic stick-slip was atom–atom distance across the contact. The simulations further demonstrated transitions between crystallographic orientation during slip events were reflected in both lateral force and current. These results confirm that the correlation between conduction and atom–atom distance previously proposed for stationary contacts can be extended to sliding contacts in the stick-slip regime.

## Abstract 2

Measuring the electrical conductivity serves as a proxy for characterizing the nanoscale contact. In this work, the correlation between sliding dynamics and current transport at single asperity sliding contact is investigated.

## Graphical abstract

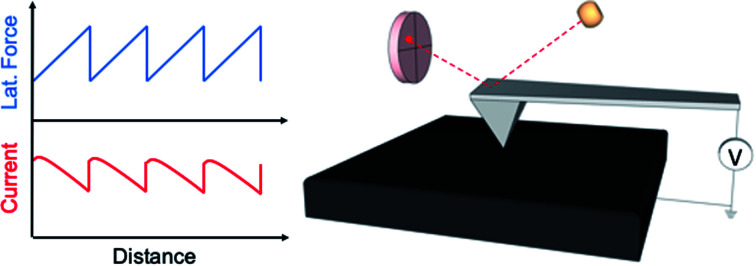

## 1. Introduction

The study of metal–metal or other electrically conductive contacts has provided foundational knowledge contributing to development of many nanoscale technologies, including nano-electromechanical switches.^1,2^ Electrically conductive contacts are also relevant to topics such as the mechanical behavior of materials,^3^ evolution of the contact formed between two bodies as they are pressed together,^4,5^ and friction between sliding surfaces.^6–10^ Such phenomena are studied using atomic force microscopy (AFM) which enables spatial, mechanical, and electrical measurements though small, well-defined contacts formed between a nanoscale AFM probe and a substrate. The electrical behavior of these contacts can be studied using conductive AFM (CAFM) where a potential bias is applied between a conductive probe and substrate and the current flow through the contact is measured.^11,12^ Studies have shown that conduction across a contact is determined by its size,^5,13,14^ surface roughness^6^ and the materials of the contacting pair.^13^ Based on these relationships, electrical current is often used as an indirect parameter to examine and interpret the structure of a contact made between two bodies.

However, studies have shown that there are limitations to using conductivity to interpret contact properties.^15^ Several CAFM investigations have focused on the relationship between current transfer and contact area, both of which should increase with pressure.^7,16–19^ However, some of these studies showed that, for very small contacts (∼50 nm or less), the expected current–contact area relationship does not always hold true.^16,17,20,21^ One explanation for this lack of correlation is that trace contamination of the contact can inhibit conduction^22^ such that the magnitude of the electrical current does not reflect the size of the contact.^16,20^ Certain environmental conditions (*e.g.* ambient conditions in which a water meniscus can form in the contact) can also break the correlation between current and contact area for nanoscale contacts.^21^ The relationship between current and contact area has been explored using simulations as well. Simulation-based studies have shown that bond distance, *e.g.* shortening or lengthening of the distance between atoms in a contact, may be a significant contributing factor for electron transport.^8,23–25^ Finally, the mechanical properties of the substrate, specifically the stiffness of a substrate material, can impact the conduction of electrons through the contact.^26^ In general, previous studies have shown that the current flow through a nanoscale contact cannot be quantitatively correlated with contact area, except for a general observation that increased contact area sometimes corresponds to increased current flow through the contact.

Beyond stationary contacts, CAFM has been used to study sliding contacts for dynamic properties such as friction. Specifically, there have been several CAFM studies where lateral force and current were measured simultaneously on atomically ordered surfaces.^7–10,27^ In these experiments, both the current and lateral force patterns exhibited the same periodicity as the substrate's atomic lattice. Recently, such studies have shown that current can be used to detect defects in the surface lattice,^9,27^ changes in conduction modes or pathways on the surface,^10^ and stacking configuration of monolayers on the substrate.^8,27^ Although these studies suggest strong correlations between conduction and the dynamic structure of the contact, the exact nature of those correlations are just beginning to be explored.

In this study, we examine the current flow through the contact formed when a nanoscale tip slides on atomically well-defined surfaces using experiments and simulations. CAFM experiments of a highly-doped diamond tip sliding on a highly-oriented pyrolytic graphite (HOPG) surface are performed. In these experiments, both the lateral force and current exhibit patterns that are characteristic of atomic stick-slip on the hexagonal lattice structure of HOPG. Also, lateral force and current are found to be inversely correlated during stick events. Molecular dynamics (MD) simulations of a simple copper-on-copper system reproduce similar trends, where the atomic stick-slip patterns have the same periodicity as the current approximated from the electrochemical potential of the atoms.^28^ The simulations are then used to explore the origins of the observed trends based on the number and positions of atoms in the contact during sliding.

## 2. Methods

### 2.1 CAFM measurements

**Fig. 1:**
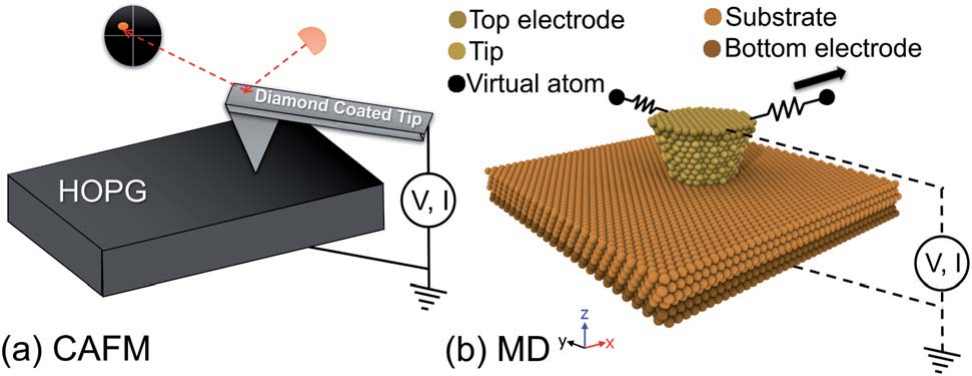
(a) Schematic of the AFM experiment and (b) snapshot of the corresponding MD simulation setup. In (b), the compliance of the system is taken into account using a spring coupled to the model tip through a virtual atom that represents the AFM cantilever. Conduction was modeled by applying a voltage bias across the contact and approximating the resulting current, as discussed in the text.

A schematic of the experimental setup is shown in Fig. 1(a). The experiment was conducted using an ultra-high vacuum (UHV) AFM (RHK Technologies 7500VT) at room temperature at a pressure of <1 × 10^−9^ Torr. A doped diamond coated cantilever (Nanosensors CDT-CONTR) with a normal bending spring constant of 0.86 N m^−1^ and lateral spring constant of 10 N m^−1^ was used to obtain all experimental data presented in this manuscript. The normal bending and torsional spring constants for the cantilever were determined using the geometric method,^29^ where the width, length and tip height of the cantilever were determined using optical microscopy and the thickness was determined using the resonance frequency of the cantilever acquired in vacuum. The conversion of volts measured by the photosensitive detector to distance was accomplished by measuring the slope of a normal force *versus* distance curve. *Post-mortem* images of the tip apex were captured using a transmission electron microscope (Tecnai F20 TEM) and circular fits to traces of the tip profile indicated a radius of 43 ± 5 nm (Fig. S1†) at the lowest asperity where the tip–sample junction was formed. Other cantilevers were also tested, including doped diamond coated silicon, PtSi coated silicon, Au coated silicon, and Pt coated silicon tips in similar experiments. With these other tips, only partial data sets were obtained, *e.g.* just a few scan lines with conductivity measurements. However, in these incomplete data sets, similar observations in terms of the lateral force, current variation during atomic stick-slip, and *I*–*V* spectra were observed.

In all experiments, the substrate was a clean HOPG sample. This surface was prepared by mechanically cleaving the sample using scotch tape under ambient environmental conditions and immediately transferring the sample into the fast entry lock of the vacuum chamber. Once the load lock was pumped to a pressure of <1 × 10^−7^ Torr, the sample was heated at 120 °C for 3 hours to remove any moisture or other contaminants. AFM topographic measurements were performed to verify the quality of the surface preparation and locate a flat atomic terrace of greater than 100 × 100 nm^2^.

Simultaneous CAFM and friction measurements were enabled by pressing the tip into the surface, applying a potential difference across the tip–sample junction, and connecting a transimpedance amplifier (FEMTO DLPCA-200) to measure the current flow through the tip–sample junction. First, *I*–*V* spectroscopy was performed to determine the resistance of the contact by sweeping the sample bias voltage from −2 to +2 V, while the cantilever tip was in contact with the sample at a given applied normal force. The contact resistance was then determined by fitting a line to the current–voltage data in the range of −0.2 V to +0.2 V where the data was close to linear. An example of this measurement is shown in Fig. S2.† The pull-off force was determined to be approximately 17 nN, and was constant throughout the subsequent series of experiments.

Following characterization of the contact resistance from *I*–*V* spectroscopy, combined CAFM and friction force microscopy (FFM) were performed. In these experiments, the tip was slid against the HOPG substrate with a constant potential bias of 1 V while maintaining a constant normal force of approximately 150 nN. During atomic stick-slip measurements, the topographic feedback was maintained at a low value to ensure the normal force was constant over a single scan frame. The lateral force, current, normal force, and topographic signals were measured simultaneously during these sliding experiments.

### 2.2 Molecular dynamics simulations

The MD simulations described a crystalline Cu (111)-terminated AFM tip apex sliding over an infinite slab of crystalline Cu (111), as illustrated in Fig. 1(b). This model system was selected because the electrical transport and frictional properties of Cu (111) are well-characterized^30–34^ and because its simplicity enabled the effects of individual parameters to be isolated and understood. The top apex was a truncated cone with the following dimensions: height 2 nm; radius of the top circle of the truncated cone 1.7 nm; and radius of the bottom circle of the truncated cone 1.0 nm. The lateral compliance of the AFM cantilever in contact with the sample was taken into account by coupling the model tip to an interaction free particle through a harmonic lateral spring (lateral stiffness of 3.2 N m^−1^) representing the cantilever.^35^ The positions of the atoms in Cu substrate were fixed and the tip was treated as a rigid body. Dynamic simulations were run in the NVT ensemble (constant number of atoms, volume and temperature) using a Nosé–Hoover thermostat.^36,37^ The thermostat was applied to maintain the temperature of the system at 0.1 K. This artificially low temperature was used to minimize thermal noise so that subtle correlations between current and friction could be detected. Periodic and fixed boundary conditions were applied in the lateral and vertical directions, respectively. The tip–substrate interactions were described by the embedded-atom method (EAM).^38^ The equations of motion were integrated with a time step of 1 fs and all simulations were carried out with the MD package LAMMPS.^39^

The simulations were performed in two stages: sliding of the tip along the surface to calculate lateral force and obtain atom trajectories; and electrical conduction calculations with atom positions obtained from the sliding simulations. To model sliding friction, the tip first was brought in contact with the substrate with a normal load of 3.5 nN and the entire system was relaxed for 0.2 ns. Then, the interaction free particle was moved with constant velocity of 2 m s^−1^ along the *x*-direction (〈1̄01〉) while the lateral force in the scanning direction was recorded. During sliding, the applied normal force was maintained at 3.5 nN while the vertical position of the tip was allowed to change. During these simulations, the atom positions were saved every 0.01 ns for use in the conduction calculations.

Electrical conduction was approximated using the EChemDID^28^ method to approximate current. First, the empirical potential used to describe tip–substrate interactions was changed from EAM to ReaxFF with potential parameters reported in ref. 40. Next, the EChemDID^28^ method was used to model the equilibration of external electrochemical potentials (voltage). Briefly, this method applies an external voltage bias (to the topmost atoms of the tip and bottom-most atoms of the substrate in Fig. 1(b)) to the reactive MD system. By equating the electrochemical potential of atoms in the system, the relative current (a unitless value that scales linearly with applied voltage) can be obtained from the combination of Ohm's law and the continuity equation under the assumption of diffusive transport without including Joule heating and electron migration effects. This technique has been successfully applied in several previous studies.^24,41–44^ Here, this method was applied to calculate current across the tip–substrate contact using atomic configurations taken from the sliding simulations.

## 3. Results and discussion

**Fig. 2:**
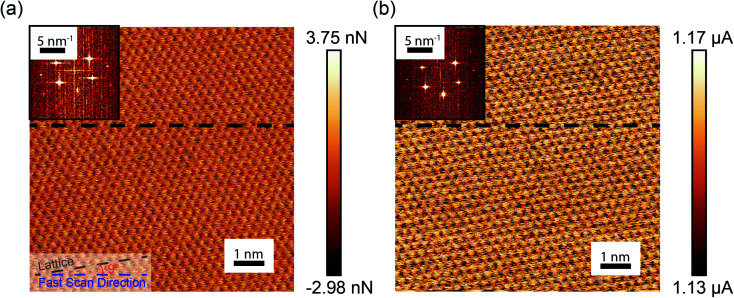
(a) Lateral force acquired over a 10 × 10 nm^2^ scan area on an HOPG surface. A hexagonal pattern is observed with periodicity corresponding to the spacing of the graphite lattice. (b) The simultaneously acquired current signal taken at 1 V. Fluctuations in the current signal match the periodicity of the atomic stick-slip seen in the lateral force image. Fourier transforms of the real-space images are shown in the insets on the top left for the lateral force and current images.

Fig. 2(a) shows a representative 10 × 10 nm^2^ lateral force image taken of the HOPG surface acquired in the UHV AFM. Fluctuations in the lateral force signal show clear lattice resolution that correspond to stick-slip friction with single slips of the diamond tip across the graphite lattice. The periodicity of the lattice was determined to be 2.5 ± 0.1 Å from the two-dimensional fast Fourier transform (FFT) shown in the upper inset of Fig. 2(a). The bottom inset of Fig. 2(a) illustrates the difference between the fast scan direction of the AFM and armchair direction of the graphene lattice is approximately 9°. Fig. 2(b) shows the simultaneous measurement of tip–sample current. Fluctuations in the current image have a periodicity of 2.5 ± 0.1 Å and a 9° difference between the fast scan direction of the AFM and the armchair direction of the graphene lattice, as determined through the FFT shown in the upper inset of Fig. 2(b). Thus, both the current and the lateral force have the same periodicity and rotation with respect to the fast scan direction of the AFM.

**Fig. 3:**
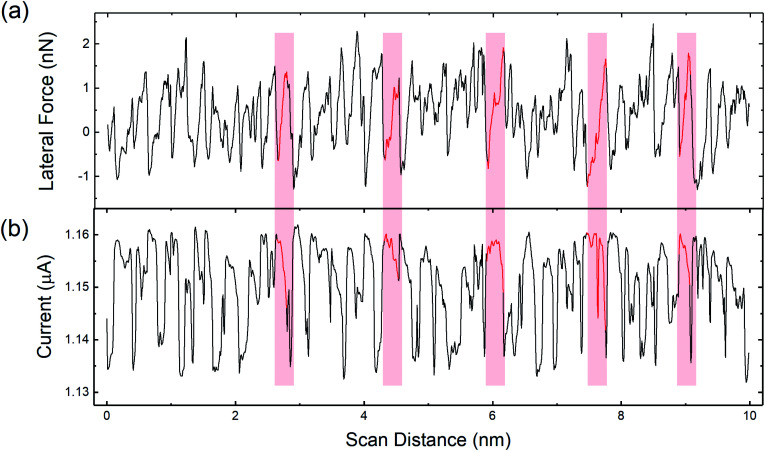
(a) Lateral force line profile acquired along the black dashed line in Fig. 2(a). (b) Current signal line profile acquired along the same line as (a), marked on Fig. 2(b). Regions highlighted in red correspond to stick events where the tip trajectory traverses the position of an atom in the HOPG lattice.

Fig. 3(a) shows the variation of the lateral force along the horizontal line in Fig. 2(a). The variation of the lateral force clearly exhibits stick-slip modulation. The periodicity of the stick-slip pattern shown here does not correspond exactly to the lattice size of the HOPG because the fast-scan direction of the AFM is not along the armchair direction of the graphite lattice. However, using the known 9° offset, stick-slip events corresponding to the AFM trajectory passing over the position of an atom in the lattice structure can be identified. Red shaded regions in Fig. 3 identify representative stick events where this is the case and the periodicity reflects the HOPG lattice. The average value of the lateral contact stiffness for these sticks was 12 ± 4 N m^−1^. For stick events not highlighted in the figure, the tip did not directly traverse atomic positions, so the stick-slip pattern does not exhibit the periodicity of the HOPG lattice. Fig. 3(b) shows the current signal variation along the same scan line as Fig. 3(a). For all five stick events highlighted in red, as the lateral force increases the current decreases. The same trends were observed in partial data sets obtained with other tips (Fig. S3†).

**Fig. 4:**
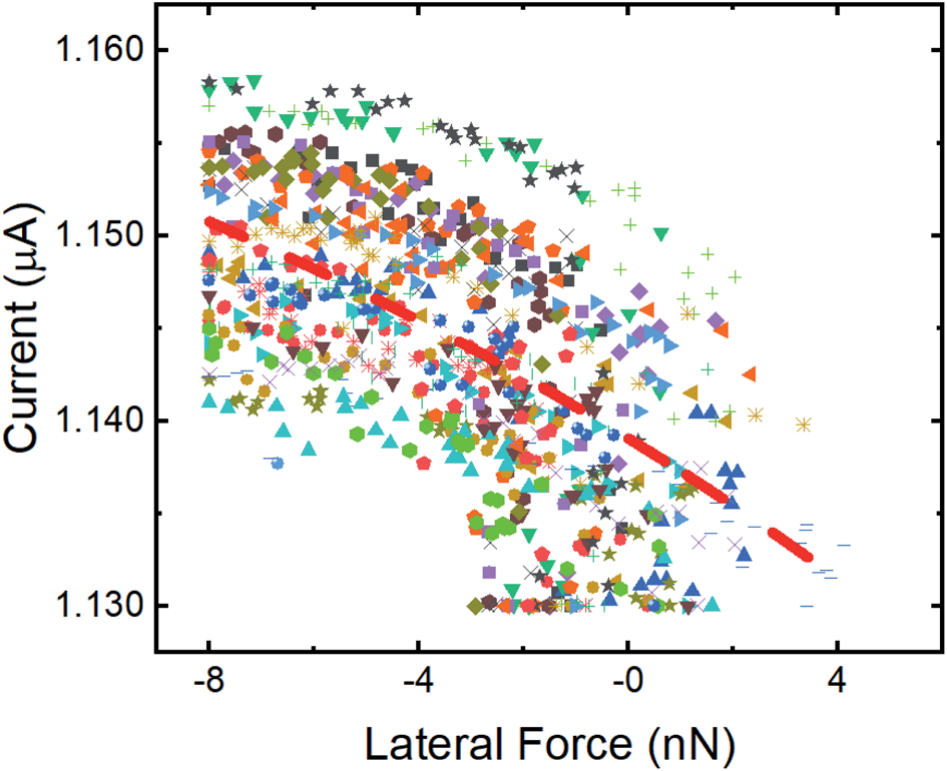
Variation of the current with lateral force during stick events where the tip trajectory traversed an atomic position from FFM/CAFM measurements. The stick data was taken from 30 lattice sites in Fig. 2 and the different stick events are identified by symbol shape and color. An exponential fit to all the data is shown as a dashed red line.

To confirm the inverse relationship between current and lateral force, 30 stick events from Fig. 2 were analyzed. The results are shown in Fig. 4 where a clear trend of decreasing current with increasing lateral force is observed, despite the scatter in the data. This scatter originates from the variation in lateral force due to thermal fluctuations at room temperature, noise inherent to the instrument, and error associated with identifying the stick events corresponding to the tip traversing the position of an atom in the graphene lattice. The data can be fit to an exponential function of the form:

1 *I*(*f*) = *A* exp(*f*/*f*_0_) + *I*_0_

where *I*(*f*) is the measured current as a function of lateral force *f*, *A* is a scaling factor, *f*_0_ is a decay constant, and *I*_0_ is a current offset. Here, *f*_0_ was found to be 23 ± 7 nN, reflecting the rate of decrease of current with increasing lateral force. The fit value of the current offset was *I*_0_ = 1.18 ± 0.02 μA, corresponding to the magnitude of the current at the onset of a stick event.

**Fig. 5:**
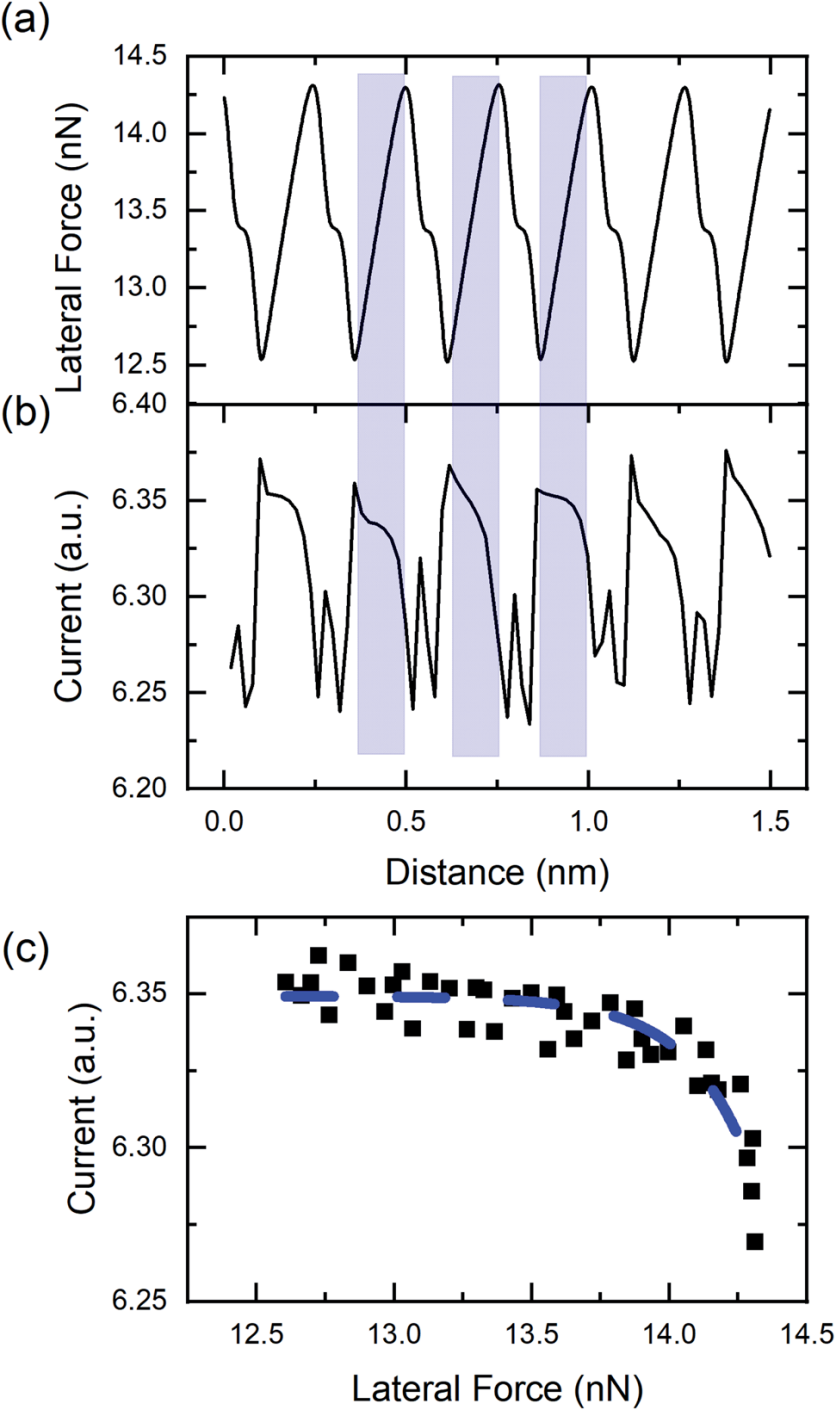
(a) Lateral force and (b) current from MD simulations of copper sliding on copper. In the stick stage, the lateral force increases while the current decreases, as highlighted by blue shaded regions on the plot. (c) Variation of the current with lateral force determined from the stick events in (a) and (b). An exponential fit is shown in the blue dashed line.

To explore the origins of the current variation during stick-slip motion, MD simulations of a simple crystalline copper sliding contact were performed. Fig. 5(a) shows the variation of the lateral force, demonstrating clear atomic stick-slip. Fig. 5(b) shows the corresponding current signal. Similar to the experimental results, the simulated current has the same periodicity as the lateral force and the current decreases as lateral force increases during the stick events. To confirm the trends were independent of material, simulations were repeated with two different orientation Cu surfaces and with the Cu (111) replaced by diamond (100). The same inverse relationship between friction and current during the stick events was observed, as shown in Fig. S4 and S5.†

Current is plotted as a function of lateral force from the simulations in Fig. 5(c), again illustrating a decrease of current with increasing lateral force. This data was fit to the exponential function in eqn (1) and the decay rate and current offset were found to be *f*_0_ = 0.23 ± 0.04 nN and *I*_0_ = 6.349 ± 0.002 a.u. The current offset in the experiment and simulation cannot be directly compared because of the arbitrary units of the simulations. However, considering the rate of change of current with lateral force, the simulation rate is two orders of magnitude smaller than that in the experiments. This reflects a faster rate of change of current with force and likely is attributable to the much larger lateral force range during stick events for the ideal, commensurate copper–copper model system. Regardless, the observation of decreasing current with increasing lateral force in both experiment and simulation, with samples having different electronic properties (*i.e.* semimetals in experiments and metal/diamond in simulations), indicates that the reported phenomenon is general and not material dependent.

The magnitude of friction measured using FFM is often correlated to contact area,^45^ where higher friction is associated with larger contact areas. Direct experimental observation of an increase in contact area during the stick phase of stick-slip motion has been observed in micron-sized contacts,^46,47^ as well as in nanoscale contacts^48,49^ and attributed to contact aging. Based on these observations, it is possible that contact size is increasing during the stick events in our measurements. However, current is expected to increase with contact area in CAFM as well.^5,18,19^ So, if contact size were increasing, both lateral force and current should increase, which is not the case in our results in Fig. 3–5. Regardless, it is possible that contact area changes are contributing to the observed trends.

**Fig. 6:**
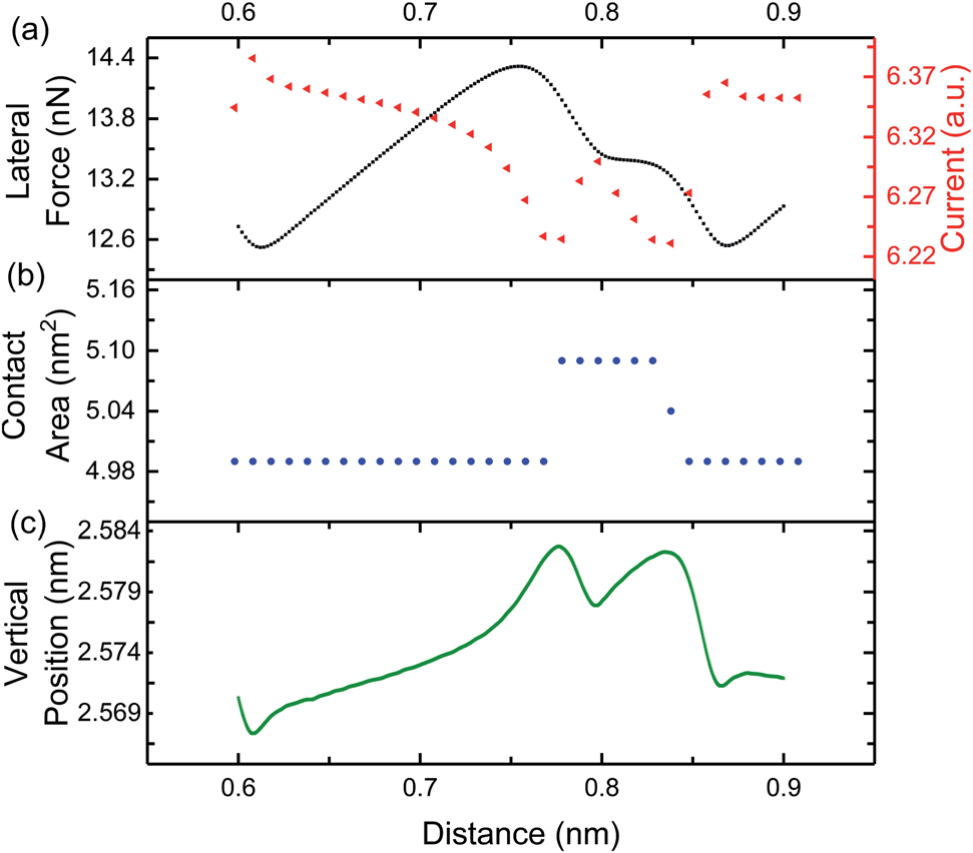
MD simulation analysis of a single stick event comparing (a) lateral force (black squares) and current (red triangles), (b) atomic contact area, and (c) the vertical position of the center of mass of the tip.

While direct measurement of contact area was not possible in our experimental setup, the real contact area could be calculated in the MD simulations. Here, the contact area was calculated by counting the number of tip atoms in contact with the substrate using a maximum atom–atom distance criteria of 0.4 nm, consistent with the cut off distance used in the EChemDID current calculation.^24^ The atomic contact area was then calculated by multiplying the number of contact atoms by an “atom area”, where atom area was approximated as the area of a circle with the atomic radius of copper.^15,50^ Theoretically, the number of contact atoms could increase due to either an increase in the apparent size of the bottom of the tip (*i.e.* the perimeter length) or through structural changes that bring more atoms in the tip close enough to atoms in the substrate to be considered in contact. In our simulations, only the latter is possible because the tip is a rigid body. Fig. 6(a) shows the lateral force and current variation over one stick-slip event and the corresponding contact area is shown in Fig. 6(b). During the stick stage, where lateral force is inversely related to current in both experiments and simulations, there is no change in contact area. Therefore, the inverse trend cannot be explained by the contact area between the tip and sample as calculated from the MD simulations.

An alternative explanation for the inverse relationship between lateral force and current in the sticking phase is the atomic distance between the atoms in the tip and the substrate. Conduction is known to increase with decreasing atom–atom distance.^8,24,34,51–53^ Further, a recent CAFM measurements of graphene on Ru(0001) showed that conduction changed with the crystallographic alignment of atoms.^8^ That study reported increased topographic height and smaller current signal in regions where the graphene/Ru(0001) had HCP stacking and lower topographic height and larger current in regions where the graphene/Ru(0001) had FCC stacking. The result was investigated using first principles calculations that showed that the trend could be attributable to be smaller atom–atom distance between the graphene and Ru(0001) substrate in the case of the HCP.^8^ We tested if this concept might be applicable to sliding contact as well.

For stick-slip motion, if the distance between atoms across the interface decreases during a stick event, an increase in current would result. Since the sample surface and tip are rigid bodies in the MD simulation, all atoms in the contact are the same distance from the substrate at each instant. However, that distance can change as the tip slides, as shown in Fig. 6(c). In this figure, higher vertical positions correspond to larger distances between tip atoms and substrate atoms across the sliding contact. We observe a monotonic increase in vertical position distance during the stick phase A similar trend was observed from simulations of sliding with two different tip–sample orientations (see Fig. S6†).

**Fig. 7:**
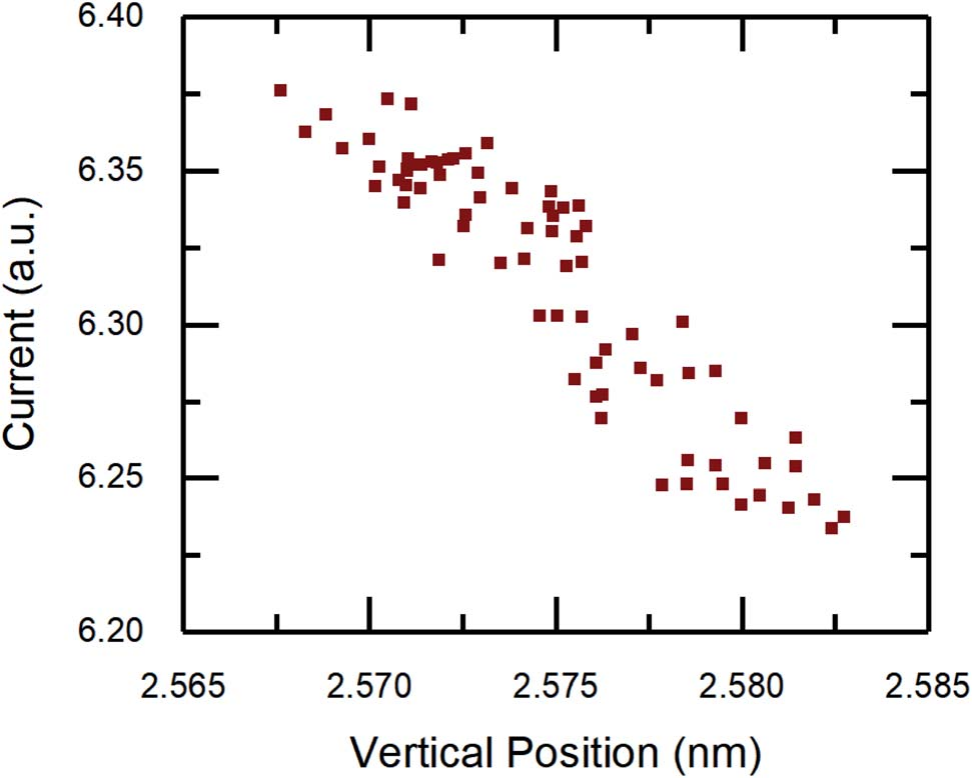
Current from the simulations as a function of tip vertical position where lower position corresponds to smaller atom–atom distance in the contact.

To confirm the relationship between current and distance, the atom–atom distance calculation was repeated for all data from the MD simulations. As shown in Fig. 7, there is a monotonic decrease of current with vertical position. Although the magnitude of the change in position is small, first principles calculations have shown that atom–atom conductance decreases exponentially with increasing distance between atoms.^8,34,51,52^ This trend is generally in agreement with an STM study of atomic scale Cu junctions in which conductance decayed exponentially with tip–sample distance.^34^ Therefore, the MD simulation results suggests that the origin of the current drop during the stick phase is an increase in tip–sample atomic distance and the corresponding conductance decay.

The hypothesis that atom–atom distance explains the inverse current–lateral force trend during stick events is supported by subtle features observed in the simulations during the slip events. Specifically, the simulated slip events exhibit transitions between FCC and HCP, characterized by shoulders in the lateral force profile^30,54^ as seen in Fig. 5(a) and 6(a). This transition from FCC to HCP corresponds to sharp peaks in the current data in Fig. 5(b) and 6(a). During this transition, when the tip is in HCP registry with the substrate, there is a local increase in the size of the contact (Fig. 6(b)) and decrease in the vertical position of the tip (Fig. 6(c)). Therefore, both increasing contact area and decreasing atom–atom distance could contribute to the local increase in current at the FCC–HCP–FCC transition. Taken together, the correlations between atomic distance and lateral force during both the stick and the slip stages indicate that the concept previously proposed for stationary contacts can be extended to stick-slip friction.

## 4. Conclusion

Simultaneous FFM and CAFM experiments with atomic lattice resolution were performed on a HOPG substrate under UHV conditions. These measurements showed the same lattice periodicity in both lateral force and current. MD simulations of a simple model system captured the FFM/CAFM experiment, where the current was approximated from the electronegativity of the atoms in the latter. In both experiments and simulations, an exponential decrease in the current during the stick phase was observed. Analysis of the contact area in the simulations indicated that the size of the contact does not change during the stick events where lateral force increases and current decreases. However, the simulations showed that tip–sample separation changed, corresponding to larger atom–atom distances across the contact and smaller current. The relationship between lateral force, atom–atom distance and current was further evaluated by analysis of FCC–HCP transitions during slip events. The results confirmed that changes in atom–atom distance can explain the observed variation in current during sliding. Although contact area change was necessarily limited in the simple model system, in practice, both contact size and atom–atom distance can affect current. We can infer from these results that an increase in tip–sample contact area will always result in an increase in tip–sample current. However, an increase in current measured during sliding does not necessarily mean that the tip–sample contact area has increased.

## Conflicts of interest

There are no conflicts to declare.

## Supplementary Material

NA-002-D0NA00414F-s001

## References

[cit1] Yao Z. J., Chen S., Eshelman S., Denniston D., Goldsmith C. (1999). J. Microelectromech. Syst..

[cit2] Goldsmith C. , Sumant A., Auciello O., Carlisle J., Zeng H., Hwang J., Palego C., Wang W., Carpick R., Adiga V., Datta A., Gudeman C., O'Brien S. and Sampath S., 2010 IEEE MTT-S International Microwave Symposium, 2010, pp. 1246–1249

[cit3] Yanson A. I., Rubio Bollinger G., Van Den Brom H. E., Agraït N., Van Ruitenbeek J. M. (1998). Nature.

[cit4] Celano U., Hantschel T., Giammaria G., Chintala R. C., Conard T., Bender H., Vandervorst W. (2015). J. Appl. Phys..

[cit5] Enachescu M., Van Den Oetelaar R. J., Carpick R. W., Ogletree D. F., Flipse C. F., Salmeron M. (1999). Tribol. Lett..

[cit6] Bowden F. P., Tabor D. (1939). Proc. R. Soc. A.

[cit7] Enachescu M., Schleef D., Ogletree D., Salmeron M. (1999). Phys. Rev. B: Condens. Matter Mater. Phys..

[cit8] Song A., Shi R., Lu H., Gao L., Li Q., Guo H., Liu Y., Zhang J., Ma Y., Tang X., Du S., Li X., Liu X., Hu Y. Z., Gao H. J., Luo J., Ma T. B. (2019). Nano Lett..

[cit9] Rodenbücher C., Bihlmayer G., Speier W., Kubacki J., Wojtyniak M., Rogala M., Wrana D., Krok F., Szot K. (2018). Nanoscale.

[cit10] Nowakowski K., Zandvliet H. J., Bampoulis P. (2019). Nano Lett..

[cit11] Houzé F., Meyer R., Schneegans O., Boyer L. (1996). Appl. Phys. Lett..

[cit12] Park J. Y., Maier S., Hendriksen B., Salmeron M. (2010). Mater. Today.

[cit13] Maxwell J. C. , A treatise on electricity and magnetism, Clarendon Press, Oxford, 1873

[cit14] Ye Z., Moon H., Lee M. H., Martini A. (2014). Tribol. Int..

[cit15] Jacobs T. D. B., Martini A. (2017). Appl. Mech. Rev..

[cit16] Vishnubhotla S. B., Chen R., Khanal S. R., Li J., Stach E. A., Martini A., Jacobs T. D. (2019). Nanotechnology.

[cit17] Vishnubhotla S. B., Chen R., Khanal S. R., Martini A., Jacobs T. D. (2019). Nanotechnology.

[cit18] O'Shea S. J., Gosvami N. N., Lim L. T. W., Hofbauer W. (2010). Jpn. J. Appl. Phys..

[cit19] Lantz M., O'Shea S., Welland M. (1997). Phys. Rev. B: Condens. Matter Mater. Phys..

[cit20] Jiang L., Weber J., Puglisi F. M., Pavan P., Larcher L., Frammelsberger W., Benstetter G., Lanza M. (2019). Materials.

[cit21] Lanza M., Porti M., Nafría M., Aymerich X., Whittaker E., Hamilton B. (2010). Rev. Sci. Instrum..

[cit22] Enachescu M., Carpick R. W., Ogletree D. F., Salmeron M. (2004). J. Appl. Phys..

[cit23] Ma B., Gong C., Wen Y., Chen R., Cho K., Shan B. (2014). J. Appl. Phys..

[cit24] Hu X., Martini A. (2017). Nanoscale.

[cit25] Srivastava S., Kino H., Joachim C. (2016). Nanoscale.

[cit26] Hu X., Lee J., Berman D., Martini A. (2018). Carbon.

[cit27] Zhang S., Gao L., Song A., Zheng X., Yao Q., Ma T., Di Z., Feng X.-Q., Li Q. (2018). Nano Lett..

[cit28] Onofrio N., Strachan A. (2015). J. Chem. Phys..

[cit29] Meyer E. , Hug H. J. and Bennewitz R., Scanning Probe Microscopy: The Lab on a Tip, Springer-Verlag, Berlin, 2004

[cit30] Sorensen M., Jacobsen K., Stoltze P. (1996). Phys. Rev. B: Condens. Matter Mater. Phys..

[cit31] Perez D., Dong Y., Martini A., Voter A. F. (2010). Phys. Rev. B: Condens. Matter Mater. Phys..

[cit32] Wolloch M., Feldbauer G., Mohn P., Redinger J., Vernes A. (2014). Phys. Rev. B: Condens. Matter Mater. Phys..

[cit33] Zhou X. S., Wei Y. M., Liu L., Chen Z. B., Tang J., Mao B. W. (2008). J. Am. Chem. Soc..

[cit34] Ternes M., González C., Lutz C. P., Hapala P., Giessibl F. J., Jelínek P., Heinrich A. J. (2011). Phys. Rev. Lett..

[cit35] Dong Y., Li Q., Martini A. (2013). J. Vac. Sci. Technol., A.

[cit36] Hoover W. G. (1985). Phys. Rev. A: At., Mol., Opt. Phys..

[cit37] Nosé S. (1984). J. Chem. Phys..

[cit38] Zhou X. W., Johnson R. A., Wadley H. N. (2004). Phys. Rev. B: Condens. Matter Mater. Phys..

[cit39] Plimpton S. (1995). J. Comput. Phys..

[cit40] Van Duin A. C., Bryantsev V. S., Diallo M. S., Goddard W. A., Rahaman O., Doren D. J., Raymand D., Hermansson K. (2010). J. Phys. Chem. A.

[cit41] Onofrio N., Guzman D., Strachan A. (2015). Nat. Mater..

[cit42] Onofrio N., Guzman D., Strachan A. (2016). Nanoscale.

[cit43] Hu X., Martini A. (2017). Nanoscale.

[cit44] Vazirisereshk M. R., Sumaiya S. A., Martini A., Baykara M. Z. (2019). Appl. Phys. Lett..

[cit45] Carpick R. W., Ogletree D. F., Salmeron M. (1999). J. Colloid Interface Sci..

[cit46] Luo Z., Song B., Han J., Yan S. (2019). Chin. Phys. B.

[cit47] Maegawa S., Suzuki A., Nakano K. (2010). Tribol. Lett..

[cit48] Li Q., Tullis T. E., Goldsby D., Carpick R. W. (2011). Nature.

[cit49] Feldmann M., Dietzel D., Tekiel A., Topple J., Grütter P., Schirmeisen A. (2016). Phys. Rev. Lett..

[cit50] Solhjoo S., Vakis A. I. (2015). Comput. Mater. Sci..

[cit51] Zotti L. A., Pérez R. (2017). Phys. Rev. B.

[cit52] Strange M., Thygesen K. S., Jacobsen K. W. (2006). Phys. Rev. B: Condens. Matter Mater. Phys..

[cit53] Chen R., Vishnubhotla S., Jacobs T., Martini A. (2019). Nanoscale.

[cit54] Martini A., Dong Y., Perez D., Voter A. F. (2009). Tribol. Lett..

