# Supplementary material for: Insights into dynamic sliding contacts from conductive atomic force microscopy
Source: Nanoscale Adv. 2020 Jul 24;2(9):4117–24. doi: 10.1039/d0na00414f (PMC9417200; doi:10.1039/d0na00414f)
Supplement: NA-002-D0NA00414F-s001 [file NA-002-D0NA00414F-s001.pdf]

Electronic Supplementary Material (ESI) for Nanoscale Advances.
This journal is © The Royal Society of Chemistry 2020

Supplementary Information for "Insights into Dynamic Sliding Contacts from Conductive Atomic Force Microscopy"

Nicholas Chan,[1] Mohammad R. Vazirisereshk,[2] Ashlie Martini,[2,*] and Philip Egberts[1,†]

[1]*Department of Mechanical and Manufacturing Engineering, University of Calgary, 2500 University Drive NW, Calgary, Alberta T2N 1N4, Canada*

[2]*School of Engineering, University of California Merced, 5200 N. Lake Road, Merced, CA, 95343, USA*

(Dated: July 13, 2020)

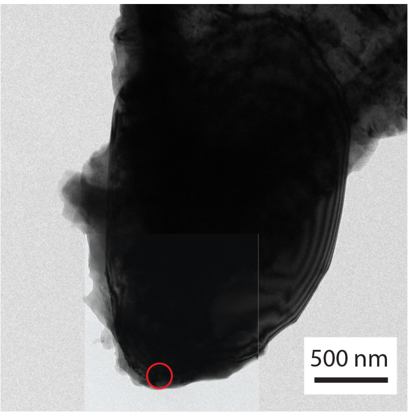

FIG. S1: Transmission electron microscopy (TEM) image of a diamond coated silicon AFM cantilever. The tip apex is rotated to capture the 22.5° angle of the AFM cantilever with respect to the sample surface. The contacting apex on the tip has been traced with a red dashed line to determine the size of the contact, which is 43 ± 5 nm.

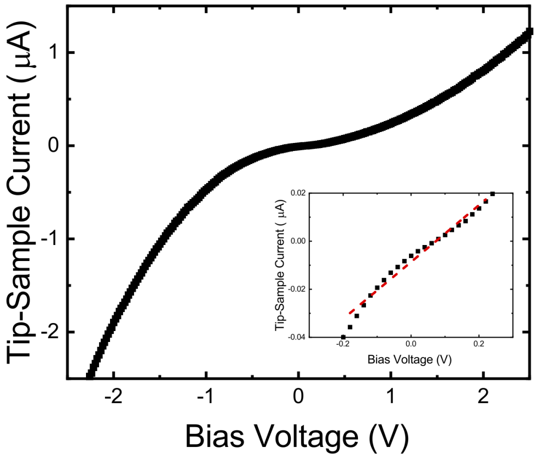

FIG. S2: The measured current flow through the tip-sample contact as a function of applied bias to the sample. The inset shows the variance from -0.2 V to +0.2V. A linear fit is shown in the inset with a dashed red line. It is clear that the current does not vary linearly over the entire range of biases examined. However, the inset in Figure S2 shows an approximately linear variation of current with sample bias over the region of -0.2 V to +0.2 V. A linear fit to this range yielded a contact resistance of 8 MΩ.

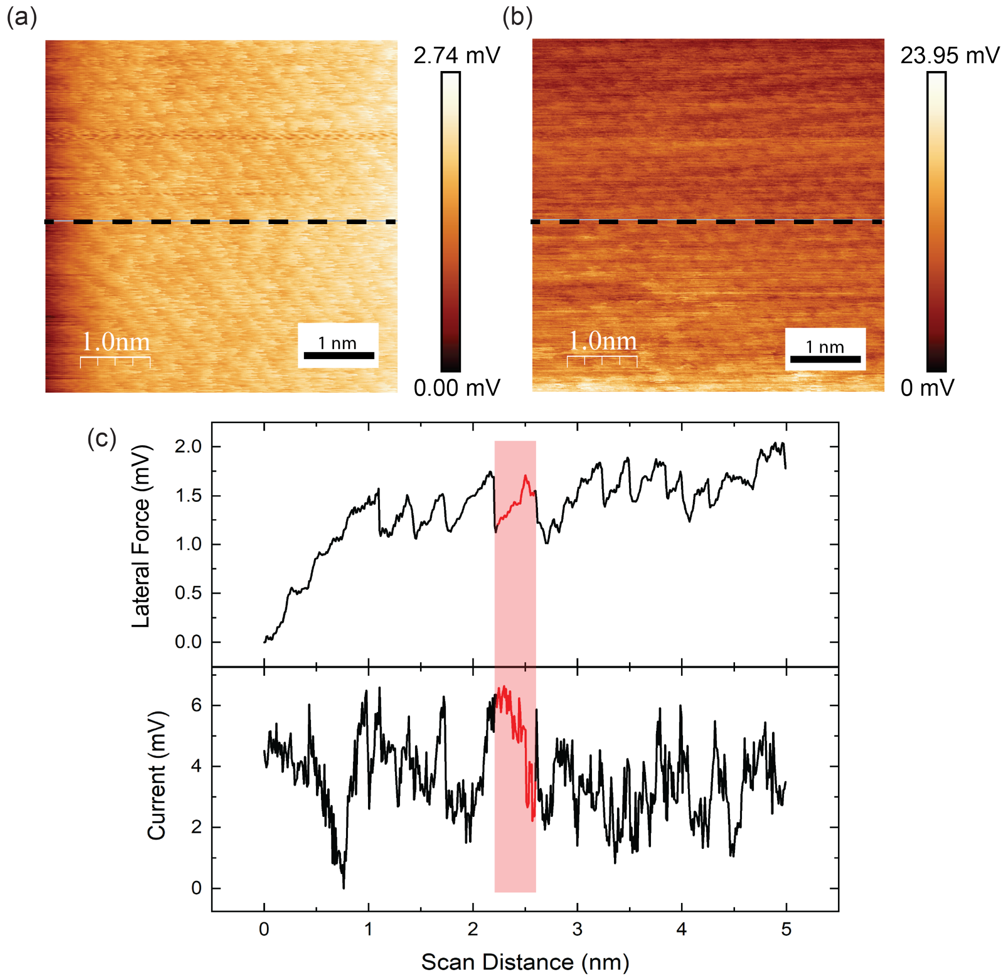

FIG. S3: Repeated measurement data from a diamond coated tip scanning on the surface. The sample bias was 1 V in this case. (a) Lateral force map, demonstrating atomic stick slip. (b) Current variation along the surface. (c) Line profiles of the lateral force and current along the dashed lines in (a) and (b), respectively. The stick event highlighted in red corresponds to the tip trajectory passing over the position of an atom in the graphite lattice. The line profiles show that, during the stick phase, the lateral force increases while the current decreases.

* amartini@ucmerced.edu
† philip.egberts@ucalgary.ca

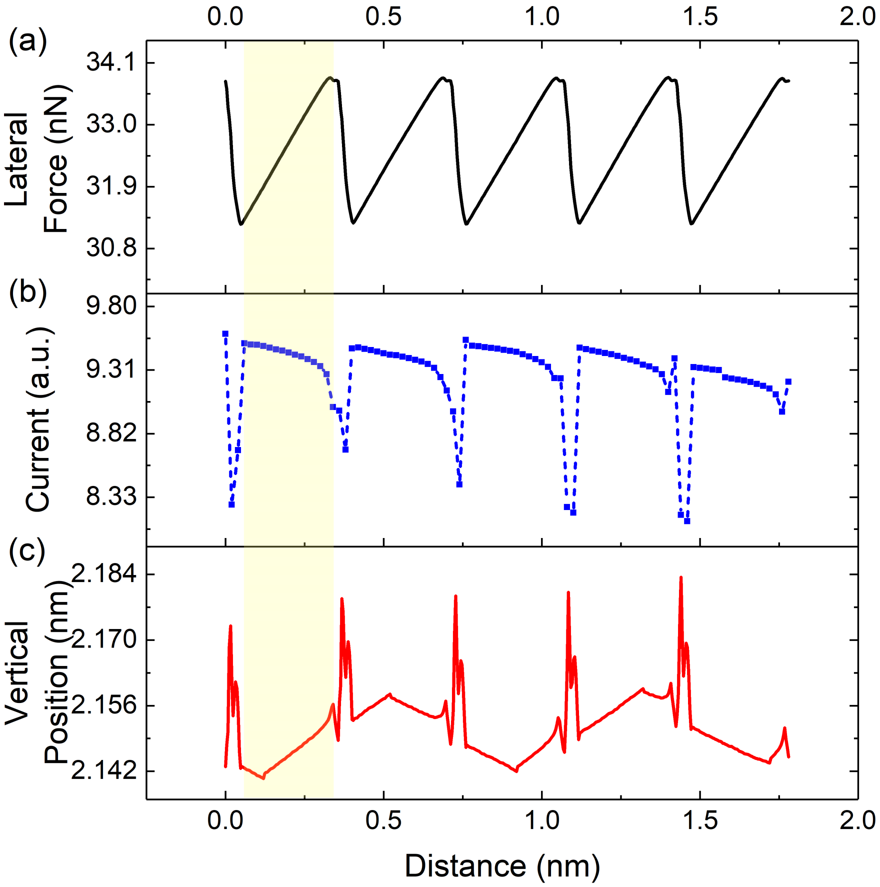

FIG. S4: (a) Lateral force, (b) calculated current, and (c) tip vertical position from MD simulations of diamond(100) terminated tip sliding on diamond(100) with a scanning speed of 4 m/s and a normal load of 0.4 nN. Lower tip vertical position corresponds to smaller atom-atom distance in the contact. Highlighted region represents a stick stage.

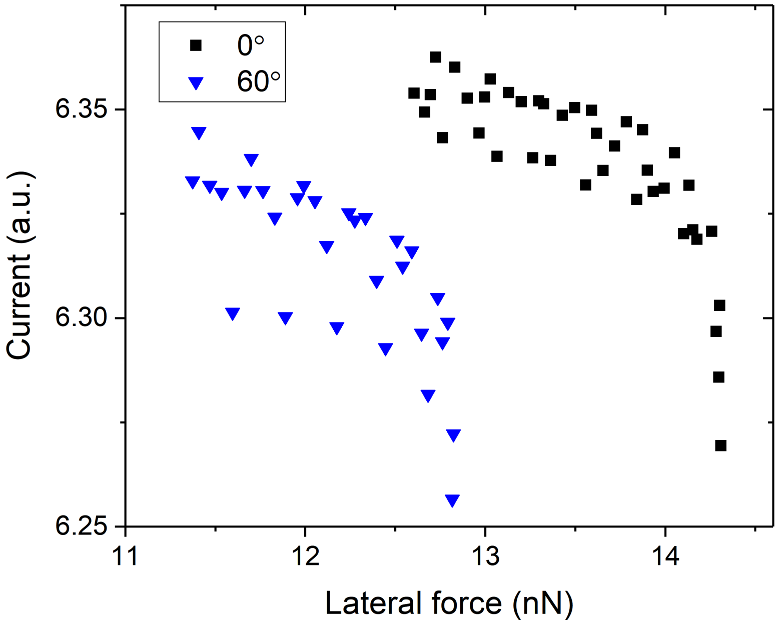

FIG. S5: Current as a function of lateral force from simulations of the Cu(111) tip at different crystallographic orientations (rotation angles) relative to the Cu (111) substrate. The sliding direction in all the cases is along the ($\langle\bar{1}01\rangle$) direction on the sample.

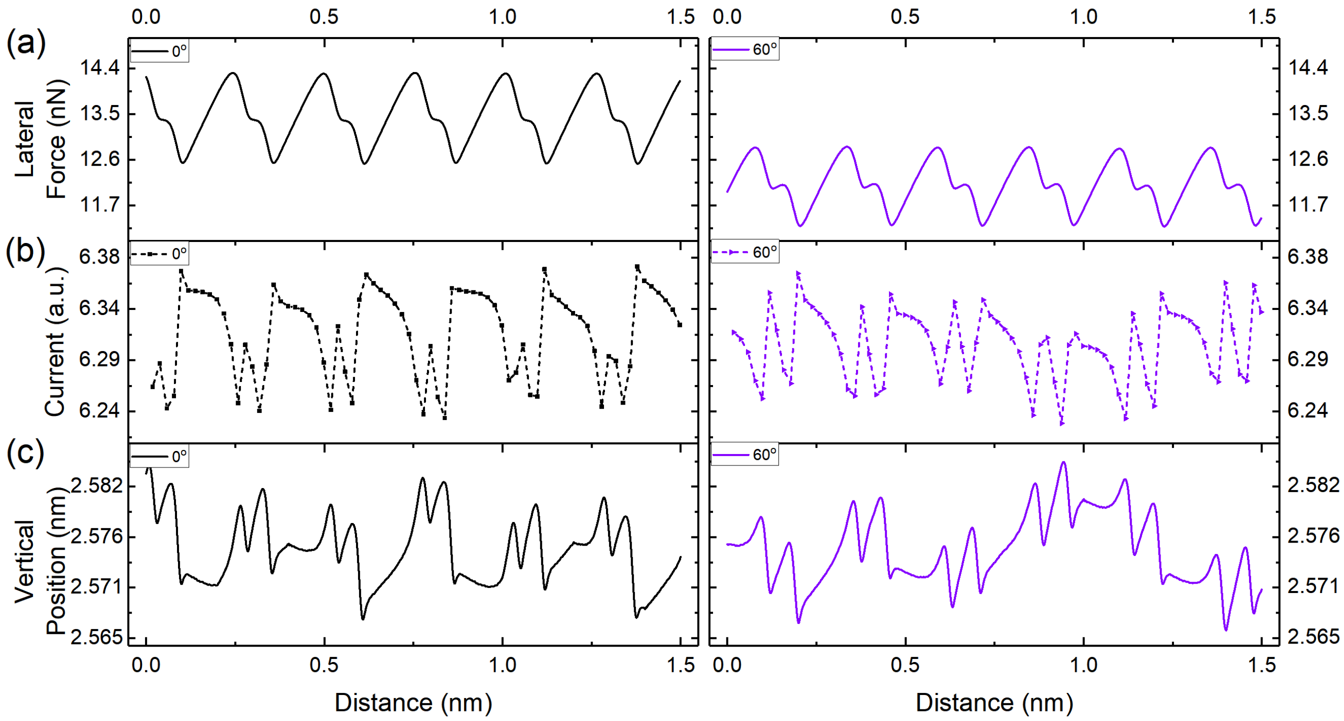

FIG. S6: MD simulation analysis of (a) lateral force, (b) current for commensurate relative tip-sample orientation angles of 0° (left) and 60° (right). and (c) vertical position of the tip center of mass for relative tip-sample orientation angles of 0° and 60° (commensurate registry). Lower vertical positions in (c) correspond to smaller atom-atom distance in the contact.
